# The Effectiveness of Surgical Thrombectomy via Below-Knee Popliteal Artery for the Treatment of Acute Limb Ischemia

**DOI:** 10.3400/avd.oa.24-00115

**Published:** 2025-01-21

**Authors:** Kentaro Kasa, Takao Ohki, Kota Shukuzawa, Soichiro Fukushima, Hirotsugu Ozawa, Makiko Omori, Yoshihiko Chono, Hiromasa Tachihara

**Affiliations:** Division of Vascular Surgery, Department of Surgery, The Jikei University School of Medicine, Tokyo, Japan

**Keywords:** acute limb ischemia, surgical revascularization, thrombectomy, below-knee popliteal artery

## Abstract

**Objectives:** Surgical thrombectomy has been established as an effective treatment for acute limb ischemia (ALI). Nevertheless, manipulation via the common femoral artery (CFA) to retrieve thrombus in the infra-popliteal artery sometimes proves less effective.

**Methods:** We retrospectively reviewed patients undergoing surgical thrombectomy for infra-inguinal ALI from January 2010 to December 2022. The primary endpoint was the rate of amputation. Secondary endpoints were technical and clinical success rates, incidence of distal embolism, and freedom from reintervention.

**Results:** A total of 35 patients underwent surgical thrombectomy where the popliteal artery or below is occluded. The CFA approach was utilized in 13, and the below-knee popliteal artery (BKPA) approach in 22. There were no differences in background between groups. The reintervention rate was lower in the BKPA group (BKPA group: 0% vs. CFA group: 30.8%; *P* = 0.01). The BKPA group showed a significantly lower incidence of distal embolism (BKPA group: 4.5% vs. CFA group: 38.5%; *P* = 0.02) and freedom from reintervention (BKPA group 100% at 12 months vs. CFA group: 68.7% at 12 months; log-rank *P* = 0.01).

**Conclusions:** The BKPA approach-first strategy for surgical thrombectomy in the management of ALI is feasible with better outcomes compared with the CFA approach.

## Introduction

Acute limb ischemia (ALI) is characterized as a condition that can lead to amputation, regardless of its cause. Without rapid diagnosis and appropriate management, the prognosis for both the limb and the patient’s life can be poor.^1^^)^ Associated with comorbidities such as cardiac and cerebrovascular diseases and ischemia-reperfusion injury, ALI has a reported mortality rate of 15%–20%.^2^^)^

Since Fogarty et al. introduced the balloon catheter in 1963,^3^^)^ balloon thrombectomy remains the gold-standard treatment for ALI.^3^^,^^4^^)^ The success of this surgical intervention, irrespective of the location of the occlusion, hinges on the complete removal of the thrombus using the Fogarty balloon catheter. Conversely, incomplete thrombus removal is associated with increased rates of re-occlusion, reintervention, and/or amputation.

Recent guidelines have highlighted the significance of ALI, designating surgical thrombectomy as the gold standard.^4^^,^^5^^)^ However, with recent advancements in endovascular treatment (EVT), many institutions now prefer EVT as their initial approach. In Japan, surgical thrombectomy remains the primary treatment for ALI due to the unavailability of modern thrombectomy devices.^6^^)^ Whether through endovascular or surgical means, one of the challenges in ALI management is preventing intraoperative embolism, which often results from incomplete thrombus removal and/or incomplete flow control.

Thrombectomy for thrombus in the unilateral iliac and/or femoral artery, performed via the common femoral artery (CFA) through an inguinal incision, is generally straightforward. However, occlusions in the popliteal artery or below pose a challenge due to the increased distance from the access site to the lesion, which reduces catheter maneuverability and makes complete thrombus removal difficult.^7^^)^ In addition, these procedures carry the risk of iatrogenic arterial dissection. Hence, direct access to the below-knee popliteal artery (BKPA) is advisable for managing thrombus in these locations.^4^^)^

The BKPA approach ensures delivery of the thrombectomy catheter and subsequent intervention into the distal superficial femoral artery (SFA), the popliteal artery, and the tibial arteries and also enables the use of appropriately sized balloons for each target vessel. This approach has been performed empirically in cases where an occluded lesion is present in the infra-popliteal artery despite the lack of reports specifically evaluating the effectiveness of the BKPA approach. Therefore, we believe that reevaluating the effectiveness of the BKPA approach is of significant value.

A pivotal case in 2017 led to a shift in our thrombectomy treatment strategy for ALI. This case, involving surgical thrombectomy via the CFA for infra-popliteal artery occlusion with subsequent reoccurrence of foot coldness due to distal embolism, highlighted the necessity of the BKPA approach-first strategy. Since this case, we have adopted thrombectomy via the BKPA as a first-line treatment for ALI with occlusions in the popliteal artery or lower. This study aimed to evaluate the initial and mid-term outcomes of the BKPA approach-first strategy in surgical thrombectomy for ALI treatment.

## Patients and Methods

The institutional review board at The Jikei University School of Medicine approved the present study (approval no. 35-210 [11839]) and waived the requirement for obtaining patient informed consent. We performed a retrospective, single-center study including patients who underwent surgical thrombectomy for ALI between January 2010 and December 2022. Patients presenting with ALI due to occlusion involving the popliteal artery or below were included in this study. Patients with iliac artery occlusion, stent graft leg occlusion following endovascular aneurysm repair (EVAR), bypass graft occlusion, CFA and/or SFA localized occlusion, popliteal artery aneurysm, and occlusion resulting from aortic dissection were excluded.

Initial assessment of ALI was performed using urgent duplex ultrasound and/or computed tomography arteriography (CTA). Before 2018, the CFA approach was predominantly utilized. The BKPA approach was employed at the surgeon’s discretion in selected cases. These cases typically involved severe and long atherosclerotic lesions in the popliteal artery or below or situations where the CFA approach failed, necessitating a switch to the BKPA approach. Since 2018, the BKPA approach has been adopted as the preferred method for cases involving occlusion of the popliteal artery or below. Our institution has been 3 or 4 attending surgeons, and surgical decision-making was left to the discretion of the attending surgeons.

### Operative procedures

For both approaches, an initial angiogram was obtained before the procedure. In the BKPA approach, a 3 Fr micro sheath was inserted in the ipsilateral CFA in a retrograde manner. In the CFA approach, the angiogram was obtained via the contralateral CFA approach. Following the angiogram, a skin incision was made in the groin to expose and encircle the CFA, the SFA, and the profunda femoris artery with vessel loops.

**Figure 1:**
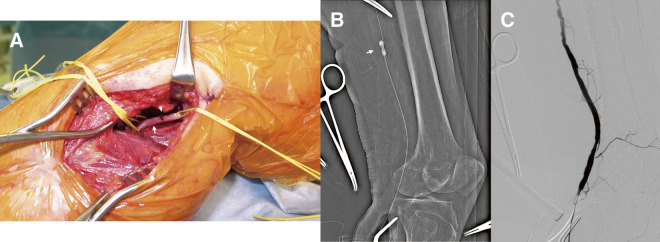
Fig. 1 The below-knee popliteal artery approach. (**A**) Below-knee popliteal artery (arrow) and thrombus (arrowhead). (**B**) Under fluoroscopic guidance, a guidewire and balloon catheter are introduced and used to remove the thrombus via thrombectomy (arrow). (**C**) With the balloon inflated at the catheter insertion site, a contrast agent is administered through the catheter’s guidewire lumen to confirm residual thrombus and arterial dissection.

In the BKPA approach, a vertical skin incision was made to separate the musculature and expose the BKPA (**Fig. 1A**). If necessary, the anterior tibial artery and the tibial peroneal trunk were also exposed and encircled with vessel loops. An arteriotomy was performed, and the thrombus present at the site of the arteriotomy was manually removed as much as possible. Under fluoroscopic guidance, a guidewire and a Fogarty balloon catheter were introduced to navigate through the thrombus, and then thrombectomy with an appropriately sized Fogarty balloon was performed under fluoroscopic guidance in the SFA, popliteal artery, and tibial vessels as required (**Fig. 1B**). With the balloon inflated in the artery at the catheter insertion site, a contrast agent was administered through the catheter’s guidewire lumen to confirm the absence of residual thrombus and arterial dissection (**Fig. 1C**).

An angiogram was also performed after the closure of the arteriotomy site to assess for residual thrombus, distal embolism, tissue perfusion, and the patency of run-off vessels. If any residual thrombi were detected, thrombectomy was repeated. Balloon angioplasty, with or without stenting, was performed in case of dissection at the thrombectomized site. Patch plasty or stenting was considered if dissection occurred at the access site and was deemed necessary. In instances where surgical thrombectomy did not successfully restore blood flow to the limb, bypass surgery was considered. Following reperfusion, if compartment syndrome developed, fasciotomy was performed on the lower leg.

### Postoperative management

In the early postoperative period, anticoagulant therapy with heparin was maintained. For patients operated on due to embolism and without significant contraindications, initiation of warfarin, targeting a long-term international normalized ratio of 2 to 3, or direct oral anticoagulant (DOAC) treatment was recommended as promptly as feasible. In cases where contraindications to warfarin existed, patients were managed with antiplatelet agents instead. For those undergoing surgery for thrombosis, antiplatelet agents were only administered unless warfarin or DOAC was already administrated for other conditions, such as atrial fibrillation and prosthetic cardiac valves.

### Endpoints and definitions of outcome

The clinical severity of ALI was classified according to the Rutherford classification system.^8^^,^^9^^)^ The primary endpoint was the rate of amputation. The secondary endpoints were technical and clinical success rates, rate of distal embolism, and freedom from reintervention.

Clinical success was defined as the resolution of symptoms related to ALI.^8^^)^ Technical success was defined as complete thrombus clearance or the restoration of flow in at least one run-off vessel through thrombectomy alone. Distal embolism was defined as slow or no flow distal to the treated site and confirmed by comparing preoperative and postoperative angiograms.

### Statistical analysis

R software, version 4.2.3, was used for statistical analysis. Continuous variables are expressed as the mean ± standard deviation or median and interquartile range (IQR). Categorical variables are expressed as counts and percentages. The Kaplan–Meier method was used to evaluate overall survival and freedom from reintervention and amputation, and the log-rank test was used to compare these variables between the early and late groups. Differences between these two groups were assessed using the χ^2^ and Fisher exact tests for categorical variables and the Student *t*-test and Mann–Whitney *U* test for continuous variables, as appropriate. Findings with a *P* value less than 0.05 were deemed statistically significant.

## Results

### Patients

From January 2010 to December 2022, 104 patients underwent surgical thrombectomy for the treatment of ALI at The Jikei University Hospital. During the study period, 38 patients with iliac artery occlusion, 12 with EVAR limb occlusion, 9 with bypass graft occlusion, 7 with CFA and/or SFA localized occlusion, 2 with popliteal artery aneurysm, and 1 with aortic dissection were excluded from the present cohort. Of these, the CFA approach was performed in 13 patients (CFA group) and the BKPA approach in 22 (BKPA group). A total of 35 patients (total mean age, 72.5 ± 12.4 years; 20% female) were included. Demographics, comorbidities, presentation, and lesion characteristics of the 35 patients are summarized in **Table 1**. There were no differences between the two groups regarding patient demographics, etiology, occluded segment, or Rutherford classification.

**Table:** Table 1 Patient characteristics

Variable	Total (n = 35)	BKPA (n = 22)	CFA (n = 13)	*P* value
Age, years	72.5 ± 12.4	74.0 ± 12.2	69.8 ± 12.1	0.346
Female, sex	7 (20.0)	4 (19.2)	3 (23.1)	1
Comorbidity				
Hypertension	25 (60.0)	17 (77.3)	8 (61.5)	0.444
Hyperlipidemia	10 (20.0)	7 (31.8)	3 (23.1)	0.709
Diabetes mellitus	9 (25.7)	5 (22.7)	4 (30.8)	0.375
Ischemic heart disease	10 (22.9)	7 (31.8)	3 (23.1)	0.709
Cerebrovascular disease	9 (25.7)	6 (27.3)	3 (23.1)	1
Chronic kidney disease	7 (20.0)	5 (22.7)	2 (15.4)	1
COPD	4 (11.4)	2 (9.1)	2 (15.4)	0.287
PAD	11 (31.4)	8 (36.4)	3 (23.1)	1
Atrial fibrillation	9 (25.7)	6 (27.3)	3 (23.1)	1
Antiplatelet use	16 (45.7)	11 (50.0)	5 (38.5)	1
Anticoagulant use	11 (31.4)	8 (36.4)	3 (23.1)	0.478
Etiology				
Embolism	16 (45.7)	12 (54.5)	4 (30.8)	0.293
Thrombosis	15 (42.9)	7 (31.8)	8 (61.5)	0.157
Others	4 (11.4)	3 (13.6)	1 (7.7)	1
Occluded segment on preoperative CTA				
SFA	17 (48.6)	8 (36.4)	9 (69.2)	0.086
Pop.A	23 (65.7)	16 (72.7)	7 (53.8)	0.292
BTKA	21 (60.0)	12 (54.5)	9 (69.2)	0.488
Rutherford classifications				
IIa	12 (34.2)	9 (40.9)	3 (23.1)	0.463
IIb	22 (62.9)	12 (54.5)	10 (76.9)	0.282
III	1 (2.9)	1 (4.5)	0 (0)	1
Duration of symptoms, hours	24 (3–240)	20 (4–240)	48 (3–240)	0.311

Data are presented as mean ± standard deviation, number (%), or median (interquartile range). COPD: chronic obstructive pulmonary disease; PAD: peripheral artery disease; CTA: computed tomography arteriography; SFA: superficial femoral artery; Pop.A: popliteal artery; BTKA: below-the-knee artery; BKPA: below-knee popliteal artery; CFA: common femoral artery

### Operative findings

Operative data are listed in **Table 2**. In the CFA group, 1 patient each was operated on under spinal and local anesthesia; all other patients were operated on under general anesthesia. The patient operated on under local anesthesia underwent a procedure using the CFA approach for distal SFA to popliteal artery occlusion, and the patient under spinal anesthesia underwent a procedure using the CFA approach for infra-popliteal artery occlusion. For 3 patients in the CFA group, the procedure was converted from the CFA approach to the BKPA approach intraoperatively.

**Table:** Table 2 Operative details

Variable	Total (n = 35)	BKPA (n = 22)	CFA (n = 13)	*P* value
Anesthesia				
General	33 (94.2)	22 (100)	11 (84.6)	0.131
Spinal	1 (2.9)	0 (0)	1 (7.7)	1
Local	1 (2.9)	0 (0)	1 (7.7)	1
Technical success	34 (97.1)	22 (100)	12 (92.3)	0.371
Additional intervention	16 (45.7)	7 (31.8)	9 (69.2)	0.04
Angioplasty	13 (37.1)	4 (18.2)	9 (69.2)	0.004
Stenting	5 (14.3)	1 (4.5)	4 (30.8)	0.052
Patch plasty	2 (5.7)	2 (9.1)	0 (0)	0.519
Bypass	3 (8.6)	2 (9.1)	1 (7.7)	1
Run-off vessel	1.8 ± 0.82	2.0 ± 0.74	1.5 ± 0.84	0.064
Distal embolism	6 (17.1)	1 (4.5)	5 (38.5)	0.02
Fasciotomy	9 (25.7)	6 (27.3)	3 (23.1)	1
Operative time, minutes	216 ± 118	229 ± 126	195 ± 102	0.415
Blood loss, mL	180 (30–1680)	230 (40–920)	150 (30–1680)	0.771
Fluoroscopy time, minutes	24 (10–77)	21 (10–59)	32 (12–77)	0.086
Contrast volume, mL	117 ± 61	102 ± 65	132 ± 47	0.190

Data are presented as mean ± standard deviation, number (%), or median (interquartile range). BKPA: below-knee popliteal artery; CFA: common femoral artery

The technical success rate for the total cohort was 97.1%, and there were no differences between the two groups (BKPA group: 100% vs. CFA group: 92.3%; *P* = 0.371). The percentage of patients who required any additional intervention during thrombectomy was higher in the CFA group than in the BKPA group (CFA group: 69.2% vs. BKPA group: 31.8%; *P* = 0.04). In terms of additional intervention details, there were more angioplasties in the CFA group (n = 9, 69.2%; *P* = 0.004). In the total cohort, conversion to bypass surgery was performed in 3 patients: 2 with embolism due to aortic wall thrombus and 1 with acute on chronic lesion in SFA. Prosthetic grafts were used in 2 patients, and a saphenous vein graft was used in 1 patient. The number of run-off vessels in the completion angiogram was 2.0 ± 0.74 in the BKPA group and 1.5 ± 0.84 in the CFA group (*P* = 0.064). The incidence of distal embolism was significantly lower in the BKPA group (CFA group: 38.5% vs. BKPA group: 4.5%; *P* = 0.02). No difference was observed in cases in which a fasciotomy was added after revascularization. No difference was found between the two groups in mean operative time (BKPA group: 229 ± 126 minutes vs. CFA group: 195 ± 102 minutes; *P* = 0.415) or in median blood loss (BKPA group: median, 230 mL; IQR, 40–920 mL vs. CFA group: median, 150 mL, IQR, 30–1680 mL; *P* = 0.771). There were no differences in intraoperative fluoroscopy time (BKPA group: median, 21 minutes; IQR, 10–59 minutes vs. CFA group: median, 32 minutes; IQR, 12–77 minutes; *P* = 0.086) and in the amount of contrast (BKPA group: 102 ± 65 mL vs. CFA group: 132 ± 47 mL; *P* = 0.190).

### Postoperative morbidity and mortality

Clinical outcomes are shown in **Table 3**. The clinical success rate for the total cohort was 88.6%, and there was no statistically significant difference in the clinical success rate (BKPA group: 95.5% vs. CFA group: 76.9%; *P* = 0.134). The median length of postoperative hospital stay was comparable between the two groups (BKPA group: median, 12 days; IQR, 5–135 days; CFA group: median, 15 days; IQR. 3–79 days; *P* = 0.383). One patient in the CFA group who developed ALI during conservative treatment for anastomotic leakage following colorectal cancer surgery suffered an ischemia–reperfusion injury after surgical thrombectomy. No other major complications occurred in the present cohort during this period. Regarding 30-day mortality, both groups were comparable (BKPA group: 4.5% vs. CFA group: 7.7%; *P* = 1). Similarly, there was no statistically significant difference in the 30-day amputation rate (BKPA group: 4.5% vs. CFA group: 23.1%; *P* = 0.134). However, the 30-day reintervention rate was significantly lower in the BKPA group than in the CFA group (BKPA group: 0% vs. CFA group: 23.1%; *P* = 0.04). Four patients died during the median follow-up of 20.1 months (IQR, 0.3–127 months) in the total cohort. In the CFA group, the patient mentioned earlier who suffered a reperfusion injury postoperatively died of multiple organ failure 4 weeks after revascularization. One patient in the BKPA group died of prosthetic graft infection at 2 weeks. In the CFA group, one patient died of primary lung cancer 5 weeks after revascularization and one died of pneumonia 4 months after surgery. Reintervention was required for four patients in the CFA group: one patient underwent redo thrombectomy via BKPA and angioplasty for re-occlusion of infra-popliteal artery on the day after surgery; one patient who had been receiving chemotherapy for lung cancer underwent a redo thrombectomy twice, at 6 and 12 days, with angioplasty for occlusion of the pedal arch and stenting for the tibial peroneal trunk, respectively; and one patient underwent stenting for edge stenosis of the previously implanted stent at 9 months, and one patient underwent angioplasty and stenting for posterior tibial artery occlusion on the day after surgery. Amputation was required for 3 patients in the CFA group: 7, 14, and 25 days after surgery, respectively.

**Table:** Table 3 Clinical outcomes

Variable	Total (n = 35)	BKPA (n = 22)	CFA (n = 13)	*P* value
Clinical success	31 (88.6)	21 (95.5)	10 (76.9)	0.134
Ischemia–reperfusion injury	1 (2.9)	0 (0)	1 (7.7)	0.371
Length of stay, days	13 (3–135)	12 (5–135)	15 (3–79)	0.383
Discharge home	26 (78.8)	18 (81.8)	8 (61.5)	0.243
30-Day mortality	2 (5.7)	1 (4.5)	1 (7.7)	1
30-Day reintervention	3 (8.6)	0 (0)	3 (23.1)	0.04
30-Day amputation	4 (11.4)	1 (4.5)	3 (23.1)	0.134
Follow-up period, months	20.1 (0.3–127)	27.5 (0.3–124)	10.9 (0.6–127)	0.506
All-cause death	4 (11.4)	1 (4.5)	3 (23.1)	0.134
Multiple organ failure	1 (2.9)	0 (0)	1 (7.7)	1
Lung cancer	1 (2.9)	0 (0)	1 (7.7)	1
Prosthetic graft infection	1 (2.9)	1 (4.5)	0 (0)	1
Pneumonia	1 (2.9)	0 (0)	1 (7.7)	1
ALI-related death	1 (2.9)	0 (0)	1 (7.7)	0.371
Reintervention	4 (11.4)	0 (0)	4 (30.8)	0.01
Thrombectomy	2 (5.7)	0 (0)	2 (15.4)	0.131
Angioplasty	3 (8.6)	0 (0)	3 (23.1)	0.04
Stenting	1 (2.9)	0 (0)	1 (7.7)	0.371
Bypass	0 (0)	0 (0)	0 (0)	N/A
Amputation	4 (11.4)	1 (4.5)	3 (23.1)	0.134

Data are presented as number (%) or median (interquartile range). ALI: acute limb ischemia; BKPA: below-knee popliteal artery; CFA: common femoral artery

**Figure 2:**
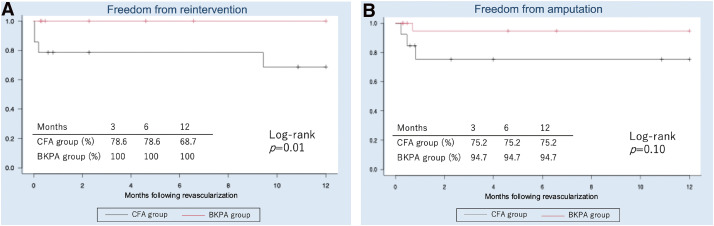
Fig. 2 Analysis of freedom from reintervention and amputation. (**A**) Freedom from reintervention was higher in the BKPA group than in the CFA group. (**B**) There was no difference in freedom from amputation between the two groups. BKPA: below-knee popliteal artery; CFA: common femoral artery

Analysis of freedom from reintervention and amputation is shown in **Fig. 2**. Freedom from reintervention rate was higher in the BKPA group than in the CFA group (BKPA group: 100% and 100% at 6 and 12 months, respectively vs. CFA group: 78.6% and 68.7% at 6 and 12 months, respectively; log-rank *P* = 0.01). There were no differences in freedom from amputation (BKPA group: 94.7% at 12 months vs. CFA group: 75.2% at 12 months; log-rank *P* = 0.10).

## Discussion

In this retrospective clinical study, we evaluated the BKPA approach-first strategy in surgical thrombectomy for ALI. Our findings indicate a significantly lower reintervention rate and incidence of distal embolism in the BKPA group (primarily BKPA approach-first), which compares favorably with the CFA group (primarily CFA approach-first strategy). In addition, the freedom from reintervention rate was significantly higher in the BKPA group. We believe that the BKPA approach, if the thrombus involves the popliteal artery or distal arteries, can improve the initial clinical outcomes in ALI patients.

Balloon thrombectomy remains the standard treatment, playing a crucial role in the surgical intervention of ALI.^3^^,^^10^^)^ In practice, despite satisfactory results in the completion of angiograms following surgical thrombectomy, a subset of patients often experience a divergent and unexpected course in the acute postoperative period. However, beyond this acute phase, patients generally achieve the anticipated good outcome. Factors contributing to early failure after surgical thrombectomy in ALI include arterial dissection associated with balloon catheter thrombectomy procedures, distal embolism, inadequate outflow in the below-knee artery, and pre-existing stenosis or occlusive lesions.^11^^)^ Hybrid treatment, combining EVT for residual thrombus after surgical thrombectomy or for underlying chronic inflow or outflow lesions, may address these early failure factors.^12^^–^^15^^)^

The BKPA approach minimizes the risk of residual clots and arterial dissection, as it allows closer and secure access to lesions in the popliteal or infra-popliteal artery. Notably, the incidence of distal embolism was significantly lower in the BKPA group than in the CFA group (4.5% vs. 38.5%; *P* = 0.02), suggesting that this approach enables more effective thrombectomy with less residual thrombus, despite the lack of studies on the effectiveness of the BKPA approach. The BKPA approach ensures delivery of the thrombectomy catheter and subsequent intervention into the distal SFA, the popliteal artery, and the tibial arteries. It also enables the use of appropriately sized balloons for each target vessel. In the current cohort, all four patients requiring reintervention were those from the CFA group. Minor residual thrombus or arterial dissections, which were not considered significant on the completion of angiogram post-procedure, were thought to have become hemodynamically significant during the postoperative course, necessitating reintervention.

The BKPA approach is potentially slightly labor intensive due to the difficulty of exposing the BKPA and the challenge of arteriotomy closure at this site. However, we observed no significant increase in operative time between the two groups. In addition, re-exposing the BKPA can be cumbersome if there is a need for future BKPA bypass surgery. Despite such theoretical concerns, prioritizing complete thrombus removal and preventing distal embolization during initial surgery to save the critically ischemic limb is of paramount importance and should be prioritized over preserving the virgin BKPA for future procedures.

Poor run-off on completion angiograms is a known predictor of adverse outcomes after ALI revascularization.^16^^)^ The presence of more outflow vessels in the completion angiogram of the BKPA group might contribute to improved outcomes in limb salvage. In our study, there are no differences in the presence of run-off vessels on completion angiograms; however, significantly reduced additional intervention after thrombectomy, especially angioplasty (BKPA group: 18.2% vs. CFA group: 69.2%; *P* = 0.004).

Although endovascular intervention is increasingly considered for ALI treatment, the unavailability of mechanical thrombectomy devices in Japan limits total EVT. Conventional catheter-directed thrombolysis poses disadvantages, including time to restore blood flow, lack of effectiveness, the need for follow-up angiograms, and bleeding risks. Often, EVT alone cannot achieve complete revascularization in ALI patients with a significant thrombus burden, making surgical thrombectomy a viable option in Japan.^7^^,^^17^^)^

The present study included several limitations, including the small number of enrolled patients, and a retrospective study with data obtained from a single institution, which may introduce selection bias in surgical decision-making. Operative decision-making was left to the discretion of the attending surgeons, and therefore, operator bias may exist. Nonetheless, given the nature and outcome of ALI, focusing on early clinical outcomes minimizes this risk.

## Conclusion

The BKPA approach-first strategy for surgical thrombectomy in the management of ALI is feasible with better outcomes compared with the CFA approach. This strategy may improve initial clinical outcomes in patients with ALI due to occlusion involving the popliteal artery or below.

## Declarations

### Acknowledgments

This work was suppprted by JSPS KAKENHI Grant Number JP24K11980.

### Disclosure statement

None of the authors have any conflicts of interest regarding this article.

### Author contributions

Study conception: KK, TO, and KS

Data collection: KK

Analysis: KK, TO, and KS

Investigation: KK, TO, and KS

Manuscript preparation: KK

Critical review and revision: all authors

Final approval of the article: all authors

Accountability for all aspects of the work: all authors.
